# Type IV secretion of *Helicobacter pylori* CagA into oral epithelial cells is prevented by the absence of CEACAM receptor expression

**DOI:** 10.1186/s13099-020-00363-8

**Published:** 2020-05-14

**Authors:** Nicole Tegtmeyer, Tabita Denisia Ghete, Verena Schmitt, Torsten Remmerbach, Maria Celeste C. Cortes, Edgardo M. Bondoc, Hans-Ludwig Graf, Bernhard B. Singer, Christian Hirsch, Steffen Backert

**Affiliations:** 1 Department of Biology, Division of Microbiology, Friedrich Alexander University Erlangen, Staudtstrasse 5, 91058 Erlangen, Germany; 2 Medical Faculty, Institute of Anatomy, University of Duisburg-Essen, Hufelandstrasse 55, 45147 Essen, Germany; 3 Division of Clinical and Experimental Oral Medicine, Department of OMF-Surgery, Leipzig University Hospital, University of Leipzig, Leipzig, Germany; 4 Center for Basic Science Research (CBSR), Research and Biotechnology (R&B), St. Luke’s Medical Center, Quezon City, Philippines; 5 Institute for Digestive and Liver Diseases, St. Luke’s Medical Center, Quezon City, Philippines; 6 Department of Oral, Maxillary, Facial and Reconstructive Plastic Surgery, University Hospital of Leipzig, Leipzig, Germany; 7 Department of Paediatric Dentistry, University School of Dental Medicine, University of Leipzig, Leipzig, Germany

**Keywords:** AGS, HN, CAL-27, BHY, CEACAM, CagA, Signaling, Type IV secretion, T4SS, Tyrosine kinases

## Abstract

**Background:**

*Helicobacter pylori* typically colonizes the human stomach, but it can occasionally be detected in the oral cavity of infected persons. Clinical outcome as a result of gastric colonization depends on presence of the pathogenicity island *cag*PAI that encodes a type-IV secretion system (T4SS) for translocation of the effector protein CagA and ADP-heptose. Upon injection into target cells, CagA is phosphorylated, which can be demonstrated by in vitro infection of the gastric epithelial cell line AGS, resulting in cell elongation. Here we investigated whether *H. pylori* can exert these responses during interaction with cells from the oral epithelium. To this purpose, three oral epithelial cell lines, HN, CAL-27 and BHY, were infected with various virulent wild-type *H. pylori* strains, and CagA delivery and ADP-heptose-mediated pro-inflammatory responses were monitored.

**Results:**

All three oral cell lines were resistant to elongation upon infection, despite similar bacterial binding capabilities. Moreover, T4SS-dependent CagA injection was absent. Resistance to CagA delivery was shown to be due to absence of CEACAM expression in these cell lines, while these surface molecules have recently been recognized as *H. pylori* T4SS receptors. Lack of CEACAM expression in HN, CAL-27 and BHY cells was overcome by genetic introduction of either CEACAM1, CEACAM5, or CEACAM6, which in each of the cell lines was proven sufficient to facilitate CagA delivery and phosphorylation upon *H. pylori* infection to levels similar to those observed with the gastric AGS cells. Pro-inflammatory responses, as measured by interleukin-8 ELISA, were induced to high levels in each cell line and CEACAM-independent.

**Conclusions:**

These results show that lack of CEACAM receptors on the surface of the oral epithelial cells was responsible for resistance to *H. pylori* CagA-dependent pathogenic activities, and confirms the important role for the T4SS-dependent interaction of these receptors with *H. pylori* in the gastric epithelium.

## Background

*Helicobacter pylori* colonizes the gastric mucosa and represents a main risk factor for gastric cancer. Approximately half of the global population is infected, and although most infections remain asymptomatic, in approximately 10–15% of infected individuals peptic ulceration occurs, and 1–2% may eventually develop gastric cancer [1, 2]. No host other than humans is known to be naturally infected by *H. pylori*, and survival outside the human body is limited. Most *H. pylori* infections initiate during early childhood and strain similarity within families suggests a parental (maternal) origin, but whether transmission occurs mainly via the oral–oral or (also) via the fecal–oral route remains subject of much debate [3–5]. Live *H. pylori* can sometimes be detected in diarrhoeic stools of infected individuals [4]. On occasion, presence of live *H. pylori* or *H. pylori* DNA has also been demonstrated in the oral cavity, mostly from specimens of dental plaque, oral mucosa, saliva or within the infected root canals of non-vital teeth [4, 6, 7]. Temporary presence of *H. pylori* in the mouth may be the result of reflux [6, 8, 9] and a meta-analysis identified an intimate association of *H. pylori* presence in the oral environment and in the stomach [10]. *H. pylori* is more difficult to eradicate from the oral cavity than from the stomach, so that oral populations may provide a source of infection to other individuals upon contact.

Colonization in the stomach depends on a number of bacterial factors, while the clinical outcome relates to presence of a chromosomally encoded pathogenicity island (PAI) carrying virulence determinants [11, 12]. This so-called *cag*PAI is only present in highly virulent strains and encodes a type-IV secretion system (T4SS) that translocates the effector protein CagA into gastric epithelial cells [13–15]. Subsequently, CagA hijacks cellular signal transduction events and causes gastric AGS cells to migrate and elongate [16]. CagA injection into host cells depends on at least 15 *cag*PAI-encoded T4SS proteins [17, 18]. The C-terminal end of CagA contains four so-called EPIYA motifs (A, B, C or D) that, once the protein is intracellularly delivered, are tyrosine-phosphorylated by host Abl and Src kinase family members [19]. The occurrence of phosphorylated CagA (CagA^PY^) is a hallmark of CagA function and an indication for its successful delivery into the host cells. *H. pylori* further expresses various adhesins on its outer membrane including BabA/B, SabA, OipA, and AlpA/B [20, 21]. Another identified adhesin, HopQ, was shown recently to bind to surface-exposed CEACAM receptors (short for carcinoembryonic antigen-related cell adhesion molecule) of the host cells. In particular, HopQ specifically interacts with the human members CEACAM1, CEACAM3, CEACAM5 and CEACAM6, and this interaction permits bacterial adhesion and is essential for delivery of CagA into a given cell [22–25]. The binding between HopQ and CEACAM can trigger CEACAM-dependent host cell signal transduction, which is a requirement for *H. pylori* colonization, T4SS functions and development of gastric pathology. However, the involved molecular mechanisms are still not fully clear.

Most of the known gastric epithelial cell lines can express CEACAM receptors and permit CagA injection [22–26]. However, whether CEACAM receptors play a role in bacterial colonization of the oral cavity has not been studied yet. Here, we investigated whether epithelial cells from the oral cavity express CEACAMs and whether they can permit CagA delivery by the T4SS of *H. pylori.* Three oral epithelial cell lines were compared, which we found were all lacking CEACAM expression and were discovered to be resistant to CagA injection. This indicates that the gastric and oral environments display different susceptibilities for T4SS effectors.

## Results

### Oral HN, CAL-27 and BHY cell lines reveal absence of cell elongation following in vitro infection with *H. pylori* strains

**Fig. 1:**
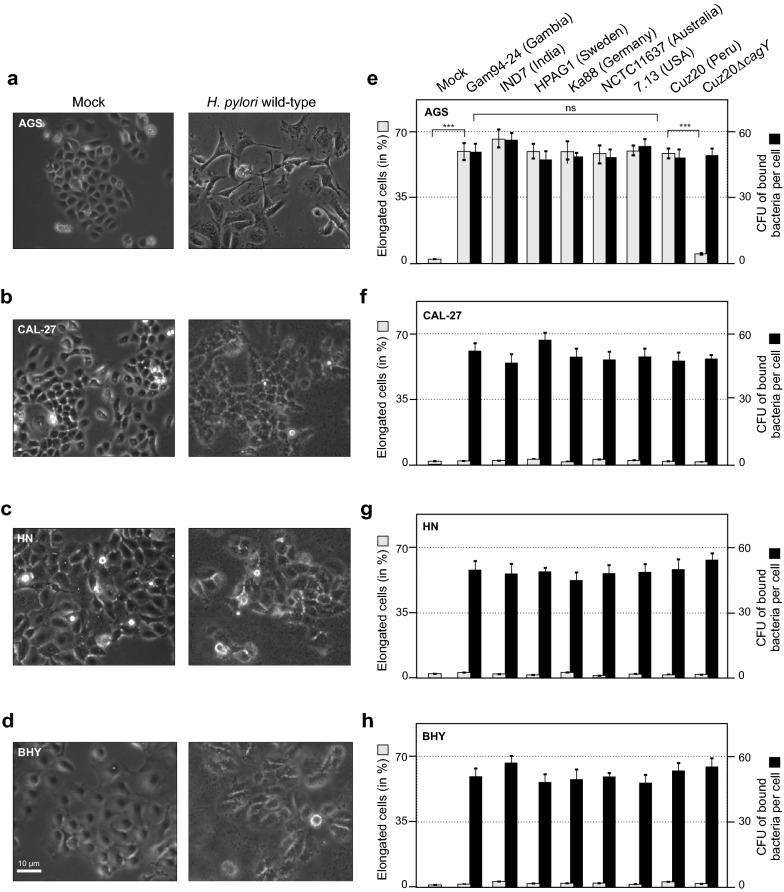
Phase contrast microscopy and quantification of elongated cells of a gastric cell line and three oral epithelial cell lines infected with *H. pylori*. Adherent cells of gastric AGS (**a**) and oral CAL-27 (**b**), HN (**c**) and BHY (**d**) cells were co-incubated with *H. pylori* wild-type strain Gam94-24 for 6 h. Panels to the left show control cells, and to the right the corresponding infected cells are shown. Cell elongation is only visible upon infection of AGS cells. Quantitative cell elongation (grey bars) and bacterial cell binding (black bars) results from triplicate experiments are shown for AGS (**e**), CAL-27 (**f**), HN (**g**) and BHY (**h**) cells following infection with the indicated seven *H. pylori* wild-type strains and a ∆*cagY* mutant of CUZ20 for 6 h (ns, not significant)

Three different cell lines originating from oral epithelial cells, HN, CAL-27 and BHY, were infected with *H. pylori* and cell morphology was compared to an infected gastric epithelial AGS cell line. Eight *H. pylori* wild-type isolates that had been isolated from various parts of the world were included. A T4SS-deficient *cagY* knockout mutant (∆*cagY*) that can no longer express CagY, which is part of the T4SS outer membrane core complex [15], was included as control. Following infection with *H. pylori* for 6 h at a multiplicity of infection (MOI) of 100, the cells were investigated by phase contrast microscopy to reveal cell elongation that is the typical outcome in infected gastric AGS cells as a result of CagA’s pathogenic activities. Figure 1a–d shows that cell elongation was absent in HN, CAL-27 and BHY cells infected with *H. pylori* strain Gam94-24 as an example. Cell elongation observed with AGS cells was quantified for all tested bacterial strains, which showed no statistically significant differences, while none of the strains caused elongation of the oral cell types (Fig. 1e–h). Bacterial binding assays demonstrated that in all cases sufficient bacteria were bound to all cell lines during infection (Fig. 1e–h). Some minor strain-dependent differences in binding capacity were observed, but these differences were not consistent with cell type (Fig. 1e–h). Thus, the lack of elongation was not a result of limited bacterial attachment to the oral epithelial cells.

### Lack of CagA phosphorylation in infected oral HN, CAL-27 and BHY cell lines

**Fig. 2:**
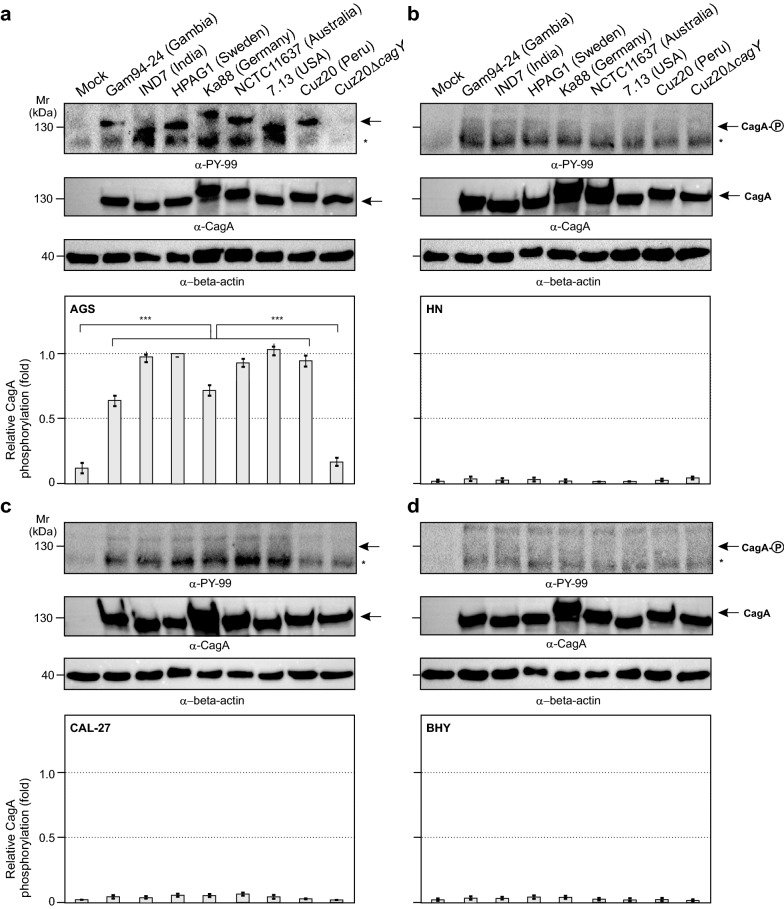
CagA internalization in host cells upon *H. pylori* infection visualized by Western blots. **a**–**d** Show Western blots stained with α-PY-99 antibodies for detection of internalized, phosphorylated CagA^PY^ on top (CagA^PY^ position indicated by an arrow), non-phosphorylated CagA detected by α-CagA antibodies as a bacterial loading control in the middle, and the β-actin protein loading control at the bottom, followed by the relative fraction of CagA^PY^ as determined by densitometry. **a** Shows AGS cells infected with the seven wild-type *H. pylori* strains plus the ∆*cagY* mutant for 6 h, and **b**–**d**) show those results for HN, CAL-27 and BHY cells. The asterisks in the α-PY-99-stained blots indicate the position of a ~ 125 kDa host cell protein

Cells of the three oral cell lines and the gastric AGS cell line were infected and harvested to produce protein lysates that were analysed by Western blots. The blots were stained with antibodies specific for CagA (α-CagA) to check proper loading with bacteria and with α-PY-99 antibodies that specifically detect intracellular CagA^PY^ [27, 28]. Antibodies recognizing β-actin were used as a protein loading control and CagA/CagA^PY^ signals were quantified to determine the level of successful CagA delivery. CagA was detected in bacteria bound to all four infected cell lines, for all tested *H. pylori* wild-type strains including the ∆*cagY* mutant, confirming that all cells had been sufficiently loaded with bacteria (Fig. 2). All wild-type *H. pylori* strains exhibited strong CagA^PY^ signals in the AGS cells as positive control, indicating CagA was successfully delivered by these strains. Densitometric quantification revealed differences in the CagA^PY^ levels obtained from infected AGS cells with the different bacterial strains, but these differences did not reach statistical significance (Fig. 2a). However, infected HN, CAL-27 or BHY cells did not result in detectable CagA^PY^ signals, suggesting internalization of CagA had not taken place (Fig. 2b–d). The lack of CagA^PY^ signals in the oral cell lines was confirmed in three independent experiments and shortening the infection time to 3 h or extending it to 12 h did not change these findings (data not shown). Although no CagA^PY^ could be detected, the α-PY-99 antibodies detected a protein band of approximately 125 kDa (smaller than CagA, indicated by an asterisk to the side of the blots) that was readily visible in all infected gastric and oral cell lines. The band was also weakly visible in lysates of uninfected oral cells and most likely represents the host cell protein vinculin [29, 30].

### Oral HN, CAL-27 and BHY cells reveal a T4SS defect associated with CagA translocation failure

**Fig. 3:**
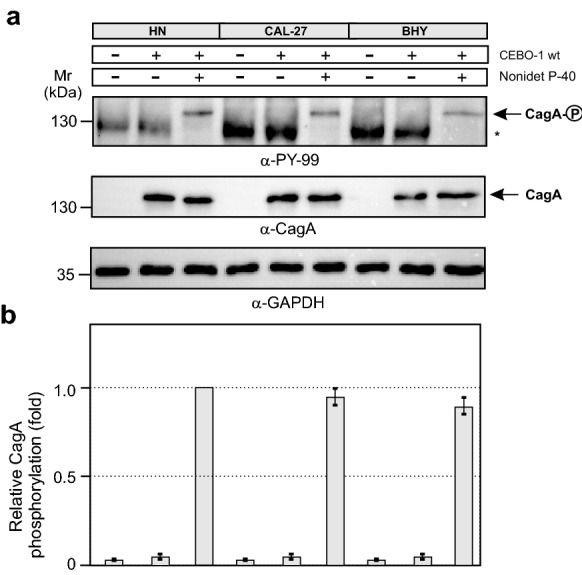
*In vitro* phosphorylation assay of CagA after infection of oral cell lines. **a** HN, CAL-27, and BHY cells were infected with indicated *H. pylori* strain for 6 h, followed by harvesting and in vitro phosphorylation assay in the presence or absence of NP-40 as described in the Experimental procedures. CagA phosphorylation was verified using Western blots. The asterisk indicates the position of a phosphorylated ~ 125 kDa host cell protein. **b** CagA^PY^ signals were quantified by densitometric measuring of band intensities

Next, we asked if the failure of CagA phosphorylation in HN, CAL-27 or BHY cells could be due to a T4SS defect or lack of activated kinases during infection. To answer this question, we infected HN, CAL-27 or BHY cells with wild-type *H. pylori* or left the cells uninfected. After 6 h, the cells were harvested and washed rigorously to eliminate non-bound bacteria, followed by centrifugation and resuspension in kinase phosphorylation buffer. In one sample per cell line, 1% Nonidet P-40 (NP-40) was added to lyse the host cells with attached *H. pylori* or left untreated to keep the cells intact, respectively. These reactions were incubated at 30 °C for 30 min, followed by harvesting and Western blotting. The resulting blots revealed that CagA from lysed cells can be profoundly phosphorylated in the presence of NP-40, but not in the non-lysed samples lacking NP-40 (Fig. 3). This implies that active kinases are present in infected HN, CAL-27 or BHY cells and lack of CagA phosphorylation is caused by compromised CagA delivery by T4SS malfunction.

### Infected oral HN, CAL-27 and BHY cells revealed high levels of IL-8 induction

**Fig. 4:**
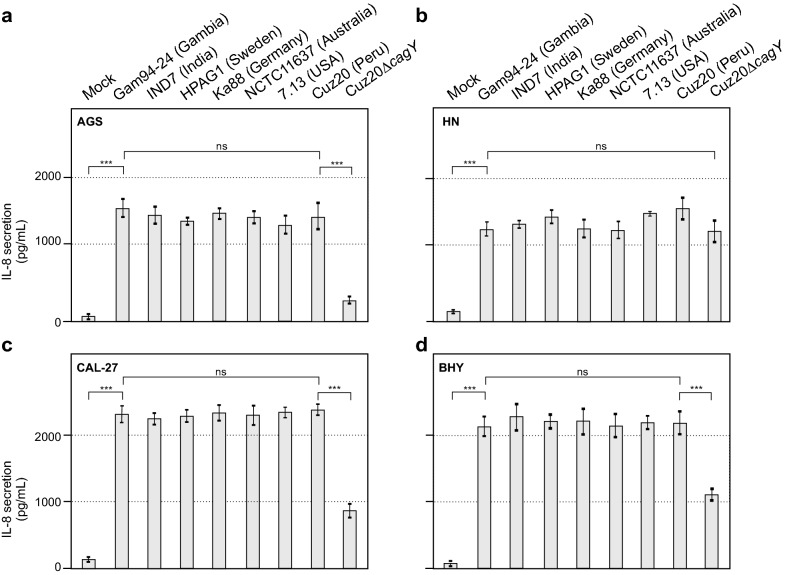
IL-8 secretion is demonstrated for all four infected cell lines. Cells were infected with all seven *H. pylori* strains and the isogenic mutant as above and after 6 h the medium was subjected to ELISA to quantify secreted IL-8, for AGS (**a**), HN (**b**), CAL-27 (**c**) and BHY (**d**) cells (ns, not significant)

In the next set of experiments, we assessed if the absence of CagA internalization following bacterial binding to HN, CAL-27 and BHY cells was accompanied by impaired induction of pro-inflammatory responses that are typically mediated by other T4SS effectors such as ADP-heptose [31]. For this purpose, the supernatants of all four infected cell lines were collected and subjected to ELISA to determine the levels of induced IL-8 production. The results show that all cell lines produced high amounts of secreted IL-8 upon infection (Fig. 4). Strain-dependent and cell type-dependent variation in the IL-8 responses was observed that was neither significant nor consistent among the four cell lines. As expected, the CagY-negative mutant revealed significantly less IL-8 secretion for AGS, CAL-127 and BHY cells (Fig. 4a, c, d), but not for HN cells (Fig. 4b). These results suggest that pro-inflammatory responses, as exemplified by IL-8 secretion, were induced in oral cells infected by *H. pylori*, even though CagA was not internalized and did not lead to cell elongation.

### The role of CEACAM members in CagA internalization by infected oral cells

**Fig. 5:**
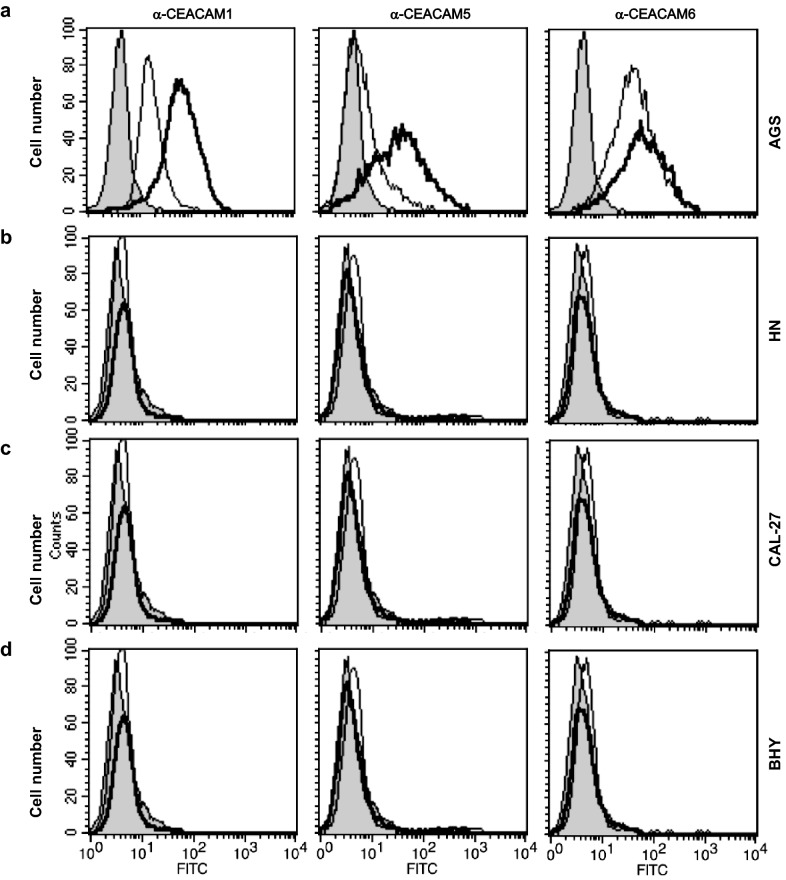
Flow cytometry examination for the presence of CEACAM1, 5 and 6 expression. CEACAM expression in AGS (**a**), HN (**b**), CAL-27 (**c**) and BHY (**d**) cells was demonstrated for non-infected cells during log phase growth (thin curves) and when in tight confluence (bold curves), stained with monoclonal antibodies specific for CEACAM1 (left), CEACAM5 (middle) and CEACAM6 (right), and with isotype-matched control antibodies (grey filled curves) followed by FITC-conjugated secondary antibody. The presented results are representative for three independent experiments

**Fig. 6:**
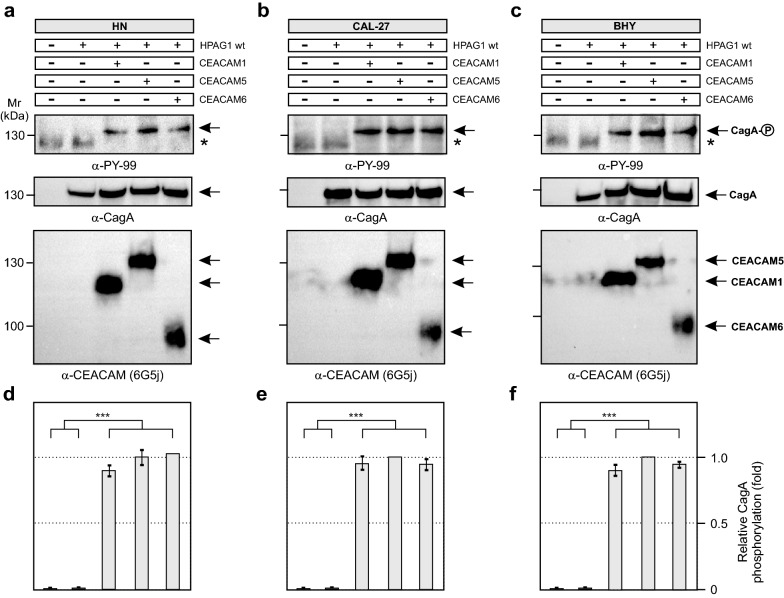
Transient expression of CEACAM1, 5 and 6 in oral epithelial cell lines restores CagA internalization. The oral cell lines HN (**a**), CAL-27 (**b**) and BHY (**c**) were transfected with CEACAM1, CEACAM5 or CEACAM6 or with an empty vector control, as indicated by the ± signs (top). The cells were then infected for 6 h with *H. pylori* strain HPAG1. Total cell lysates were subjected to Western blotting stained with specific monoclonal antibodies to identify expression of CEACAM1, 5 or 6, respectively (blots shown on top) and with α-PY99 and α-CagA to demonstrate CagA internalization in the oral cells as a result of CEACAM expression. **d**–**f** CagA internalization was quantified by band intensities from three independent experiments

To elucidate why CagA was not delivered into the oral cells, we investigated whether these cell types expressed CEACAM receptors. Previous Western blotting studies had revealed that AGS cells expressed three T4SS-relevant CEACAM members, sized approximately 120, 180 and 90 kDa, corresponding to CEACAM members 1, 5 and 6, respectively [22, 23, 26]. Here, we subjected uninfected HN, CAL-27 and BHY cells to flow cytometry and used monoclonal antibodies specific for detection of each of these three CEACAM members (Fig. 5). The results confirmed that AGS cells indeed express all three CEACAMs (Fig. 5a), while the HN, CAL-27 and BHY cells did not produce any of those CEACAM receptors (Fig. 5b–d). This lack of CEACAM expression may, in part or completely, account for the lack of CagA internalization in these oral epithelial cell lines. To investigate this further, the oral cells were transfected with plasmid constructs expressing either CEACAM1, 5 or 6 as previously described [26] and these transfected cells were infected with *H. pylori*. Expression of the three CEACAM receptors by the transfected cell lines was confirmed by Western blotting using monoclonal antibody 6G5j recognizing CEACAM1, 5 and 6 (Fig. 6). When these cells expressing a single CEACAM type were infected with *H. pylori*, CagA^PY^ signals were clearly visible in cell lysates following staining with PY-99 antibodies (Fig. 6, top). The intensity of the obtained CagA^PY^ signals demonstrated that there were no significant differences for the three CEACAM types resulting in CagA internalization, in either of the three cell lines, and that the fraction of intracellular CagA was similar to that obtained with AGS cells (*cf*. Fig. 2). This demonstrates that the reason CagA was not internalized by the oral cell lines (as per Fig. 2) is due to their lack of CEACAM expression. Interestingly, CEACAM expression in oral cells followed by infection with *H. pylori* did not rescue the elongation phenotype, suggesting the existence of yet unknown AGS gastric epithelial cell-specific features that are absent in HN, CAL-27 and BHY cell lines.

## Discussion

During gastric infection, *H. pylori* interacts with its host by means of various factors, including VacA, CagA and T4SS-injected effector molecules. Their roles in bacterial colonization, persistence and development of gastric disease have been studied in detail [1, 2, 32, 33]. This has led to a model of host–pathogen interactions that starts with the activity of BabA and SabA adhesins that initiate and sustain contact to the host cell’s Lewis antigens [20, 21]. The bacterial T4SS then binds to factors on the surface of the host cell to enable intercellular delivery of CagA. A role of integrin-β_1_, phosphatidylserine and cholesterol has been implicated in T4SS-dependent CagA injection [17, 34–39], but of crucial importance are the CEACAM receptors and their bacterial ligand HopQ, as they need to interact for full functionality of the *H. pylori* T4SS [21–26]. We have recently proposed that HopQ binding to CEACAM molecules may bring the T4SS-pilus in the optimal position and distance to allow delivery of CagA [26]. Most of those observations were made with the gastric cell line AGS. In the present study, we investigated the interactions between *H. pylori* and three oral epithelial cell lines (HN, CAL-27 and BHY), which confirmed the dependence on CEACAM receptors for CagA intracellular delivery. We found that these oral cell lines did not express CEACAM receptors naturally, while infecting the cells in vitro with T4SS-positive wild-type strains of *H. pylori* did not result in CagA intracellular delivery or pathogenic effects. Intracellular CagA delivery, however, could be rescued by genetic introduction of single representatives of T4SS-relevant CEACAM members, which now resulted in internalized and phosphorylated CagA. Obviously, the lack of CEACAM members is not the only difference between the studied gastric and oral epithelial cell lines, which explains why restoration of CagA delivery and phosphorylation in the oral cells by means of CEACAM expression did not result in cell elongation upon *H. pylori* infection that is typical for infected gastric AGS cells (Fig. 1). The reason for this is currently under investigation.

Similar to the oral cell lines analyzed here, several previous in vivo studies showed that CEACAMs are not or only barely expressed in the healthy oral epithelium and other cell types of the oral mucosa [40, 41]. However, a spatiotemporal CEACAM expression during the embryonic development of the oral epithelium or as a consequence of oral infection with certain bacteria such as *Fusobacter spp.* in connection with inflammatory reactions has been reported [42, 43]. Increased CEACAM expression was also described for other pathological scenarios in the oral cavity such as in oral lichen planus patients or in smokers [44, 45]. CEACAM1 was also shown to be overexpressed in oral tumors [40]. Ultimately, the pathological induction of CEACAM expression in the oral cavity may further exacerbate the situation in the presence of *H. pylori*, since CagA could then be translocated into the cells leading to a further deterioration in health condition of the patient. Unfortunately, there are no in vivo data yet to support this idea. Therefore, studies addressing this interesting hypothesis should be subject of future studies.

Oral epithelial cell lines are not the only cell types resistant to CagA translocation and phosphorylation, as this was also demonstrated for other non-gastric cell lines (e.g. HEK293, CHO) and gastric cell line MKN28, which also happens to completely lack CEACAM expression [22–24, 46]. Similar to the findings reported here for oral cell lines, sensitivity to CagA can be introduced in these cell lines by prior transfection with CEACAM1, 5 or 6 [22, 23]. Recently, we further demonstrated that duodenal AZ-521 cells do not permit CagA translocation, despite producing integrin-β_1_ [26, 47] phosphatidylserine [48], and cholesterol (our unpublished data). Again, these cells completely lack expression of CEACAM members, but in contrast to the observations made with the abovementioned cell types and the oral cell lines, only transfection of AZ-521 cells with CEACAM1 or CEACAM5 induced CagA sensitivity, while expression of CEACAM6 had no effect [26]. In contrast, HeLa cells (a cervical adenocarcinoma cell line) lack expression of CEACAM1, 3, 5 and 6, but these cells are sensitive to delivery and phosphorylation of CagA [49, 50]. In this case, CagA internalization may result from an alternative mechanism, as it was further shown that CagA could be internalized by HeLa cells when delivered by a *hopQ* deletion mutant [50]. This contrasts with observations made with AGS, Caco-2, MKN45 or other CEACAM-expressing gastrointestinal cell lines that all require bacterial HopQ to interact with CEACAM receptors for profound T4SS-dependent CagA delivery [22–24, 46].

The strong induction of pro-inflammatory responses through means of IL-8 secretion by all infected oral epithelial cell lines is surprising. Usually, T4SS-dependent CagA translocation and inflammation are coupled processes that occur at the same time [51, 52]. However, it was recently shown that HopQ, which can bind to a specific subset of CEACAMs, promotes canonical NF-κB activation and IL-8 secretion in AGS and NCI-N87 cells, but not in HeLa cells, which are devoid of the CEACAMs [50]. Thus, it seems that *H. pylori* can activate CEACAM-dependent and CEACAM-independent pathways of NF-κB activation and IL-8 secretion depending on the cell line. The same maybe true for strong IL-8 induction in the CEACAM-deficient HN, CAL-27 and BHY cell lines. It also appears remarkable that while *H. pylori*-triggered IL-8 secretion was T4SS-dependent in CAL-27 and BHY cells, this was not the case in HN cells. In the latter cell line, the T4SS-deficient Cuz∆*cagY* mutant produced similar high IL-8 levels compared to wild-type infections. The reason for this difference is unclear. We propose that a yet unknown non-canonical T4SS-independent NF-κB pathway is induced in infected HN cells. It will be interesting to investigate this specific signal transduction cascade in forthcoming studies.

## Conclusions

The work performed here clearly demonstrates that cell lines obtained from the oral epithelium are resistant to T4SS-dependent injection of CagA by *H. pylori*, similarly to most CEACAM-negative cells (except for HeLa) and that this resistance is overcome by genetic introduction and expression of single CEACAM types. These findings have implications for potential oral colonization by *H. pylori*. The oral cavity is actually considered as a temporary habitat during transmission of gastric *H. pylori* from person to person [1–7]. This assumption is in agreement with our present finding that a major docking site of *H. pylori* is missing on the oral epithelium. Furthermore, the absence of CEACAMs on the surface of healthy oral epithelial cells would result in the lack of T4SS-related pathogenic effects through injected CagA of otherwise highly virulent strains. However, since an immune response in these cells would still be elicited (as demonstrated here by IL-8 production) it cannot be excluded that presence of *H. pylori* in the oral cavity may lead to local inflammatory reactions, similar to their activities in the stomach. This idea needs to be investigated in future research.

## Methods

### Cell lines and tissue cultures

The oral cell lines were all from squamous cell carcinomas. Cell line HN (ACC 417; DSMZ Braunschweig/Germany) was derived from a patient with an invasive tumour of the soft palate, although the cell line was isolated from a tumour of the cervical lymph node 7 years after initial treatment of the primary tumour. The CAL-27 cell line (ATCC CRL-2095™) was from a tumour of the tongue, and the BHY cell line (ACC 404, DSMZ) was derived from a squamous cell carcinoma of the lower alveolus that had invaded to the mandibular bone and muscle layer of the oral floor.

These cell lines, together with the human gastric adenocarcinoma cell line AGS (ATCC CRL-1739™) were cultured in RPMI 1640 medium with 10% heat-inactivated foetal calf serum (Gibco, Paisley, UK), 2 mM l-glutamine (Invitrogen, Karlsruhe, Germany) and 1% antibiotic/antimycotic solution (Sigma-Aldrich) at 37 °C under 5% CO_2_. Cells were subcultured at a ratio of 1:3–5 every 2–3 days at 70 to 90% confluence. All cells were maintained in 75 cm^2^ tissue culture flasks and seeded into 6-well plates (Greiner-Bio-One, Germany) to reach approximately 3.5 × 10^5^ cells per well (about 80% confluency) for infection experiments.

The HN, CAL-27 and BHY cells were transfected and then cultivated for 48 h with human CEACAM1, CEACAM5 and CEACAM6 expression constructs or an empty vector control using previously described experimental procedure [23, 26].

### *H. pylori* strains and culture conditions

The *H. pylori* strains utilized in this report, strains Gam94-24, IND7, HPAG1, Ka88, NCTC11637, 7.13 and Cuz20, all contained the *cag*PAI [27, 28, 53, 54]. The strain CEBO-1 was cultivated from a patient from the Phillipines with gastritis (this study). An isogenic ∆*cagY* mutant of Cuz20 was produced as previously described [26] and was selected and grown in presence of 4 μg/mL chloramphenicol. All *H. pylori* strains were cultivated from glycerol stocks on GC agar plates containing 5% horse serum, 5 μg/mL trimethoprim, 10 μg/mL vancomycin and 1 μg/mL nystatin [30]. The agar plates were incubated in jars in an atmosphere of 85% N_2_, 10% CO_2_ and 5% O_2_ (Oxoid, Wesel/Germany) at 37 °C for 2 days.

### Experimental infection of cells and bacterial binding assays

Bacteria (*H. pylori* wild-type strains and the isogenic ∆*cagY* Cuz20 mutant) were harvested from agar plates using sterile cotton swabs (Carl Roth, Karlsruhe/Germany) and resuspended in phosphate buffered saline, pH 7.4 (PBS) at concentrations of identical OD_600_. The exact numbers of colony-forming units (CFU) was established by plating serial dilutions on agar plates [55]. Cells were washed with once with PBS and culture medium devoid of antibiotics or antimycotics and *H. pylori* was added at an MOI of 100 (approximately 3.5 × 10^7^ CFU/well) in all experiments. The cells were then incubated for 6 h as reported previously [56]. An uninfected mock control was included where PBS lacking bacteria was added.

For binding assays, after 6 h of incubation the cells were washed three times with 1 mL of pre-warmed culture medium without antibiotics to eliminate non-attached bacteria, after which the cells were lysed by incubation with 1 mL 0.1% saponin in PBS at 37 °C for 15 min [57]. After collection serial dilutions of the cell lysates were cultivated on GC agar plates for quantification of cell-bound *H. pylori* CFU.

### In vitro CagA phosphorylation assay

After infecting approximately 3.5 × 10^5^ HN, CAL-27 or BHY cells with wild-type *H. pylori* for 6 h, the cells were harvested with a cell scraper in ice-cold kinase buffer (150 mM NaCl, 25 mM HEPES, pH 7.0, 5 mM dithiothreitol, 10 mM MgCl_2_, 1 mM Na_3_VO_4_, 1 × Complete™ proteinase inhibitor mix) followed by two washing steps and centrifugation at 500×*g* to remove unbound bacteria [26, 58]. Using a pipette, each pellet was then gently mixed with 200 μL fresh kinase buffer or kinase buffer containing 1% NP-40 (Sigma-Aldrich) for cell lysis. ATP (1 mM) was added to start the in vitro phosphorylation reaction for 30 min at 30 °C. Uninfected cells were used as negative control. The reactions were terminated by adding 60 μL 4× reducing SDS-PAGE buffer (10% SDS, 10% mercaptoethanol, 200 mM Tris–HCl, pH 6.8, 0.4% bromphenol blue, 40% glycerol) followed by 5 min boiling and Western blotting analyses.

### Phase contrast microscopy

Phase contrast images of infected cells were obtained with a Leica DMI4000B microscope. Cell elongation as a result of infection was quantified using criteria for elongation as described previously [51, 59] to be scored as elongated thin cell protrusions with sizes between 20 and 70 µm had to be present. Protrusions shorter than 10 µm were ignored. The experiments were performed in triplicate. All images were read in a blinded manner.

### Flow cytometry for detection of CEACAM expression

To analyse CEACAM expression, cells from all four cell lines were harvested during log phase growth and after reaching confluence. The cells were stained with monoclonal antibodies α-CEACAM1, α-CEACAM5, α-CEACAM6 and isotype-matched control antibodies, followed by FITC-conjugated secondary antibody. Subsequently, samples were analysed by flow cytometry as previously described [26].

### Western blotting

Infected cells (produced as described above) were harvested, mixed with an equal volume of 2 × SDS-PAGE buffer and lysed at 100 °C for 5 min. The cell lysates were then separated by 6–12% gradient SDS-PAGE, and Western blotted onto PVDF membranes (Immobilon-P, Merck Millipore). Prior to staining the membranes were blocked with 25 mM Tris–HCl pH 7.4, 140 mM NaCl, 0.1% Tween-20, 3% BSA for 1 h at 20 °C. CagA protein was detected with rabbit polyclonal α-CagA antibody (# HPP-5003-9, Austral Biologicals, San Ramon/USA) and α-rabbit polyvalent goat secondary antibody (# 31462, Thermo Fisher Scientific, Massachusetts/USA). Phosphorylated CagA (CagA^PY^) was detected by α-PY-99 mouse antibodies and β-actin with monoclonal mouse antibodies (# A5441, Sigma-Aldrich), followed by α-mouse goat antibodies (# 31446). Monoclonal antibody α-CEACAM (6G5j) recognizes CEACAM1, CEACAM5 and CEACAM6 and has been described elsewhere [23]. Secondary antibodies were conjugated with horseradish peroxidase (Thermo Fisher Scientific, Massachusetts/USA) and detected with the ECL Plus chemiluminescence Western Blot kit (GE Healthcare).

Densitometric analysis for quantification of band intensity was performed with Image Lab software (BioRad, Munich/Germany) and following normalization of band intensities, the ratio of CagA^PY^ over CagA signal was plotted for each lane [26].

### ELISA for detection of IL-8

All four cell lines were infected with the seven *H. pylori* strains and the deletion mutant as described above. After 6 h the cell medium was collected and subjected to ELISA to quantify secreted IL-8, using a commercially available assay kit (Becton–Dickinson, Germany).

### Statistical data analysis

Every experiment was performed in triplicate and statistical significance was calculated with GraphPad Prism statistical software (version 8.0). Data were evaluated via one-way ANOVA followed by Tukey’s multiple comparison test. Significant difference was defined by *p ≤ 0.05, **p ≤ 0.01, and ***p ≤ 0.001.

## Data Availability

The datasets used and/or analysed during the current study are available from the corresponding author on reasonable request.

## Abbreviations

CagA^PY^: Phosphorylated CagA
CEACAM: Carcinoembryonic antigen-related cell adhesion molecule
CFU: Colony forming units
MOI: Multiplicity of infection
NP-40: Nonidet P-40
PAI: Pathogenicity island
PBS: Phophate buffered saline
T4SS: Type-IV secretion system
α-CagA: Antibodies specific for CagA
